# The Potential of Allelochemicals from Microalgae for Biopesticides

**DOI:** 10.3390/plants12091896

**Published:** 2023-05-06

**Authors:** Livia Marques Casanova, Andrew Macrae, Jacqueline Elis de Souza, Athayde Neves Junior, Alane Beatriz Vermelho

**Affiliations:** 1 Biotechnology Center-Bioinovar, Institute of Microbiology Paulo de Goes, Universidade Federal do Rio de Janeiro (UFRJ), Rio de Janeiro 21941-902, RJ, Brazil; 2 Sustainable Biotechnology and Microbial Bioinformatics Laboratory, Institute of Microbiology Paulo de Goes, Universidade Federal do Rio de Janeiro (UFRJ), Rio de Janeiro 21941-902, RJ, Brazil

**Keywords:** bioherbicide, biocide, biopesticide, allelochemical, microalgae

## Abstract

Improvements in agricultural productivity are required to meet the demand of a growing world population. Phytopathogens, weeds, and insects are challenges to agricultural production. The toxicity and widespread application of persistent synthetic pesticides poses a major threat to human and ecosystem health. Therefore, sustainable strategies to control pests are essential for agricultural systems to enhance productivity within a green paradigm. Allelochemicals are a less persistent, safer, and friendly alternative to efficient pest management, as they tend to be less toxic to non-target organisms and more easily degradable. Microalgae produce a great variety of allelopathic substances whose biocontrol potential against weeds, insects, and phytopathogenic fungi and bacteria has received much attention. This review provides up-to-date information and a critical perspective on allelochemicals from microalgae and their potential as biopesticides.

## 1. Introduction

Allelopathy is an ecological phenomenon in which allelochemicals are produced under certain environmental conditions. The term ‘allelopathy’ was introduced by Molisch in 1937 and originated from the Greek words ‘allelon’ (mutual) and “pathos” (harm). The term means one organism influences others through a chemical mode [1].

Allelochemicals are toxic secondary metabolites produced by plants, microalgae, bacteria, and fungi that influence the development of other organisms. These substances can be harmful to humans, other microalgae, and organisms, including phytoplankton, animals, and submerged macrophytes. Photosynthesis and enzyme activity inhibition, damage to cell structures, and other physiological alterations such as inhibition of respiration, protein synthesis, and gene expression have been observed [2,3].

Allelopathy is much studied in plants, but allelochemicals started to be reviewed in microalgae by Inderjit and Dakshini in 1994 [2,4]. Microalgae are a widely diversified group of prokaryotic and eukaryotic photosynthetic microorganisms. More than 50,000 microalgae species are distributed in rivers, lakes, oceans, and even in terrestrial ecosystems. Cyanobacteria are prokaryotic microalgae in which membrane-bound organelles such as plastids and mitochondria are missing, while eukaryotic microalgae rely on these structures to control cellular functions [5,6]. Many common microalgae taxa are included in the latter group, such as Chlorophyta (green algae) and Bacillariophyta (diatoms) [5,6]. Pyrrophyta (dinoflagellates) are mostly microplankton, they are eukaryotic and live in aquatic environments, be it salt, brackish or fresh water. Around half of all dinoflagellates are photosynthetic, while the other half are heterotrophic; some live as mixotrophic and obligate symbionts/parasites. They are usually reported as microalgae; indeed, the most toxic microalgae are dinoflagellates [7] The non-algal dinoflagellates are primarily predators of other microorganisms or parasites of animals or giant protists [8].

One of the fields of study for allelochemicals in microalgae began with harmful algal blooms (HABs). HABs are a consequence of an ecosystem imbalance in marine and freshwater systems worldwide [9]. HABs affect water usage, including fisheries, water resources, and recruitment. The poisoning caused by the allelochemicals affects different organisms and leads to economic losses [10]. The use of allelochemicals as biocontrol agents has been suggested as a means of improving microalgae cultivation and controlling contamination [11]. An example of a mode of action is the generation of reactive oxygen species (ROS). *Nostoc spongiaeforme* produces a pigment named nostocine A; this pigment is highly cytotoxic for several microalgae. The compound accelerates ROS formation in *Chlorella reinhardtii.* Nostocine A penetrates the target and is reduced by NADPH; when oxygen levels are high, reduced nostocine A is oxidated, generating superoxide radical anion (O^2−^) that may cause cytotoxicity [12].

**Figure 1:**
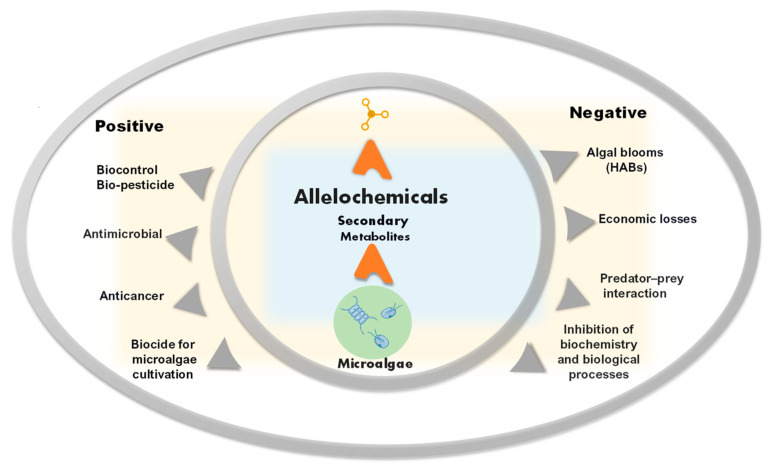
Schematic representation of positive and negative aspects of allelochemicals from microalgae.

The bioactivity of allelochemicals and their toxic or beneficial effects have also been the focus of research in medicine and agriculture [13], and for their anticancer [14] and antimicrobial properties (Figure 1) [15].

Toxic compounds that are used to protect crops, and to control weeds, insects and disease, are known as pesticides. The extensive and widespread use of synthetic pesticides, however, poses several long-term health and environmental risks. Often, pesticides are also toxic to humans and animals, resulting in carcinogenicity, teratogenicity, and the disruption of hormonal systems. Pesticides that persist in the environment for years result in the contamination of water and food sources [16,17]. Another challenging problem for agriculture is the emergence of pesticide resistance in target species [18]. In a scenario of increasing pollution on the planet and of climate changes triggering environmental problems, it is urgent that sustainable, green methods for the biocontrol of pests are found. Environmentally-friendly allelochemicals from microalgae have been described as bioherbicides. These compounds arise as an alternative source of natural biocides to reduce the use of toxic synthetic biocides/herbicides [19].

This review aims to examine allelopathy in microalgae, focusing on the potential applications of allelochemicals as herbicides, insecticides, and in their action against phytopathogens.

## 2. Allelopathy in Microalgae

As mentioned previously, the term allelopathy was originally used to describe biomolecules produced by plants. However, it is now openly used with other organisms and has been studied in microalgae [19,20]. Microalgae organisms produce numerous secondary metabolites, including fatty acids, alkaloids, amino acids, and peptides, which are investigated and characterized as natural products by chemists and described as novel bio compounds. In the environment, these molecules are secreted for communication, defence, and adaptation purposes [21]. More knowledge is needed in order to understand the complexity of these bioactive chemicals and their interaction with and effect on ecosystems [19,22].

A recent review paper showed that *Microcystis*, *Nodularia*, *Karenia*, *Alexandrium (A. catenella*, *A. fundyense*, and others species), *Skeletonema*, *Chlorella* (*C. vulgaris*, *C. sorokiniana*, *C. ellipsoidea*), *Chlamydomonas*, and *Dunaliella* displayed allelopathic activity against other microalgae and cyanobacteria [23]. One strategy to study the allelopathic effects of different microalgae agents is to analyse the biochemicals present in mono- and co-cultures [24]. Wang et al. [24] observed that the dinoflagellate *Scrippsiella* acuminata had its growth significantly inhibited when in co-cultivation and/or in the presence of exudates (cell-free and sonicated-cell filtrates) from three diatoms (*Chaetoceros curvisetus*, *Phaeodactylum tricornutum* and *Skeletonema dohrnii*), suggesting the diatoms developed significant competitive advantage over other antagonists. Furthermore, the authors detected several types of volatile organic compounds (VOCs) in the sonicated cell-free filtrates. Polyunsaturated aldehydes (PUAs) were found in all three diatoms that showed inhibitory effects on the growth of *S. acuminata* and the diatoms themselves. Generally, the production of microalgae VOCs is influenced by environmental conditions, such as nutrient availability, temperature, and light incidence [25,26].

Studies show that allelochemicals of microalgae have either a positive or negative impact on other organisms. Those which can cause inhibitory effects are suitable for application as biopesticides, and as a new source of antimicrobials or bio-herbicide agents [19,23], especially because these biocompounds present increased target specificity [27]. The liberation of species-specific allelochemicals is desired in agriculture and is an important focus for future studies because they can be responsible for modulating the ecosystem under favourable conditions to an allelopathic microalgae. An example of this was observed in a study where, by alternating the nutrients conditions and balance of nitrogen and phosphorus sources, the allelopathic interaction between strains of *Anabaena* spp. and *Microcystis* spp. seemed to control their relative abundance and their dominance in that habitat [28]. 

An interesting point to remark upon here is that one of several new applications of microalgae is as a bioenergy source and an alternative to fossil fuels [29,30,31]. Nevertheless, one of the elements that industry must be alert to is the accumulation of allelopathic molecules released into the water by microalgae themselves. These compounds, when in high concentrations due to high biomass, along with other biomolecules can trigger positive or negative effects on other cells, leading to auto-stimulation or self-inhibition [32,33,34]. Lu and colleagues (2020) described studies that focused on water reuse for microalgae cultivation, and stated that several factors, including allelopathy mechanisms, cause cell damage, aggregation, and programmed cell death (PCD) (negative effects) or, on the other hand, the liberation of growth regulators and removal of growth inhibitors (positive effects). Bacterial agents can have a significant influence on allelopathy affecting microalgae biomass tanks [33,35].

Some allelopathic molecules have been characterized in the scientific literature as having a role in algal community growth regulation. Satake et al. [36] described a 19 membered ring-structured molecule called alexandrolide, which was isolated from dinoflagellate *Alexandrium catenella* and inhibited diatoms (*Skeletonema costatum* and *Chattonella antiqua*) but did not inhibit other dinoflagellates. Marennine, a blue-green polyphenolic pigment produced by diatom *Haslea ostrearia*, was described as being responsible for inhibiting the growth of other diatom species and self-stimulating the producer species in oyster-ponds [37]. By elucidating the properties of these compounds, more studies can be performed to apply these chemicals for industrial and human purposes, such as in additives in agricultural technology [38].

## 3. Allelochemicals from Microalgae

Microalgae are the source of a remarkable diversity of biologically active compounds, which can be involved in allelopathic interactions. The main chemical classes of microalgal allelochemicals are: alkaloids, fatty acids and derivatives, polyketides, peptides, phenolics, and terpenoids. 

### 3.1. Alkaloids

**Figure 2:**
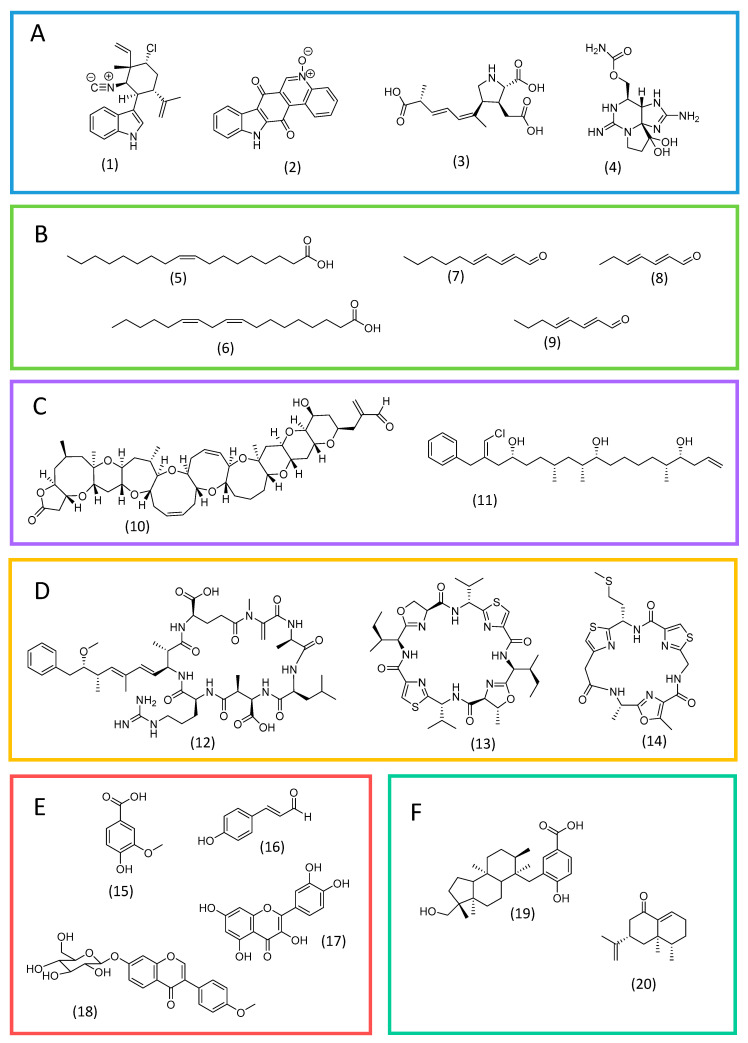
Examples of allelochemicals from microalgae. Alkaloids (**A**): 12-*epi*-hapalindole E isonitrile (**1**), calothrixin A (**2**), domoic acid (**3**), and saxitoxin (**4**). Fatty acids and derivatives (**B**): oleic acid (**5**), linoleic acid (**6**), 2*E*,4*E*-decadienal (**7**), 2*E*,4*E*-heptadienal (**8**), and 2*E*,4*E*-octadienal (**9**). Polyketides (**C**): brevetoxin A (**10**) and trichophycin A (**11**). Peptides (**D**): microcystin-LR (**12**), patellamide A (**13**), and tenuecyclamide C (**14**). Phenolic substances (**E**): vanillic acid (**15**), p-coumaric acid (**16**), quercetin (**17**) and ononin (**18**). Terpenoids (**F**): comnostin A (**19**) and eremophilone (**20**).

Alkaloids are a prominent class of allelochemicals with biocidal properties. They consist of nitrogen-containing heterocyclic substances, in which the nitrogen atom is usually derived from an amino acid [39,40]. Alkaloids have been reported mainly in cyanobacteria and dinoflagellates. In the former, indole alkaloids are the most frequent. For instance, 12-*epi*-hapalindole E isonitrile (Figure 2A; **1**), from *Fischerella* sp., is an indole alkaloid with activity against green microalgae and other microorganisms [41]. Another example is the toxin calothrixin A (Figure 2A; **2**) from *Calothrix* sp [41].

**Figure 3:**
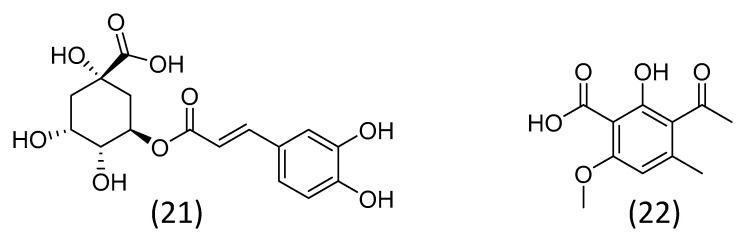
Phenolic substances active against phytopathogens: chlorogenic acid (**21**) and 3-acetyl-2-hydroxy-6-methoxy-4-methyl benzoic acid (**22**).

**Figure 4:**
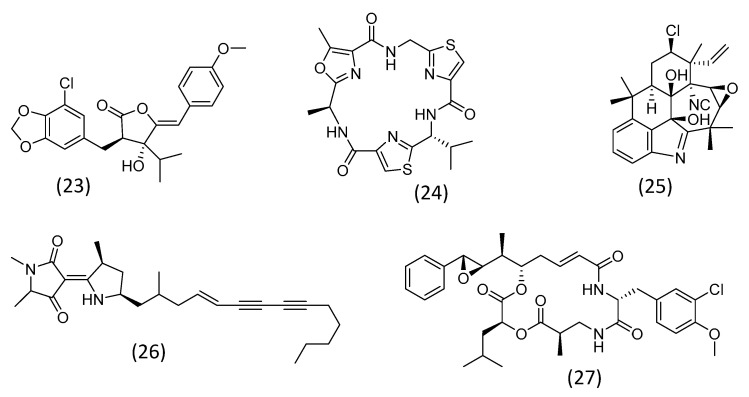
Microalgal substances with herbicidal potential: cyanobacterin (**23**), nostocyclamide (**24**), ambiguine D isonitrile (**25**), fischerellin A (**26**), and cryptophycin A (**27**).

Domoic acid (Figure 2A; **3**) is a well-known biotoxin from the bloom-generating diatom *Pseudo-nitzschia* sp. which has a pyrrolidine skeleton derived from glutamic acid [42,43,44]. Another example of an alkaloid from diatoms is saxitoxin (Figure 2A; **4**), a potent neurotoxin with a complex skeleton whose nitrogen atoms are derived from arginine. It has been reported in cyanobacterial species as well [45,46,47,48].

### 3.2. Fatty acids and Derivatives

Extracellular free fatty acids from microalgae have a possible allelopathic effect, controlling the growth of other microalgae and harmful organisms. Green microalgae, such as *Chlorella* sp. and *Botryococcus braunii*, are reported to secrete mixtures of common fatty acids (e.g., oleic and linoleic acids; Figure 2B) under certain conditions, which may favor their dominance [49,50]. Additionally, unsaturated fatty acids are reported to possess antimicrobial activity [51]. 

Substances derived from fatty acids can also act as allelochemicals. Polyunsaturated fatty acids (PUFAs), after various oxidative transformations, originate polyunsaturated aldehydes (PUAS) (e.g., 2*E*,4*E-*decadienal, 2*E*,4*E*-heptadienal, and 2*E*,4*E*-octadienal; Figure 2B), which play a role in communication and chemical defense in diatoms [11,39,52]. They exert a deleterious effect on grazers and competing microalgal species, and stimulate organic-matter-recycling bacteria [52]. 

### 3.3. Polyketides

Polyketides are a large group of structurally diverse substances biosynthesized from carboxylic acid precursors through repeated cycles of condensation, reduction and dehydration reactions catalyzed by multifunctional enzymatic complexes called polyketide synthases (PKS). These substances are found mainly in dinoflagellates and cyanobacteria and are frequently associated with harmful blooms [39,53]. Examples include maitotoxin from the dinoflagellate *Gambierdiscus* sp., the largest and most toxic known polyketide [54,55], and also brevetoxins from *Karenia brevis* (e.g., brevetoxin A; Figure 2C; **10**), a dinoflagellate associated with “red tides” [56]. Another remarkable example are trichophycins (e.g., trichophycin A; Figure 2C; **11**), linear chlorinated polyketides from cyanobacteria from the genus *Trichodesmium*, which produce frequent blooms in the Pacific Ocean [57,58]. 

### 3.4. Peptides

A significant amount of cyanobacterial allelochemicals are peptides. Most of them belong to the group of non-ribosomal peptides, which are biosynthesized by multifunctional enzyme complexes, the non-ribosomal peptide synthases (NRPS). NPRS are similar to PKS and assemble sequentially common amino acids but also unusual amino acids [12,39,59]. For example, microcystins are cyclic non-ribosomal peptides produced by cyanobacteria of the genus *Anabaena*, *Microcystis*, *Planktothrix*, *Oscillatoria*, and others. They are toxic for microalgae, aquatic plants, and for mammals and birds as well [12,60]. Microcystin-LR (Figure 2D; **12**) is the most frequent of them [60]. 

Ribosomal peptides also occur in cyanobacteria. For instance, cyanobactins are small cyclic peptides formed solely from proteinogenic amino acids and reported in many genus, such as *Anabaena*, *Lyngbya*, *Microcystis*, *Nostoc*, and *Trichodesmium* [59,61]. They possess antimicrobial activity and probably have the function of inhibiting the growth of competing and harmful microorganisms. Patellamide A, from *Prochloron*, and tenuecyclamide C, from *Trichodesmium erythraeum*, are representatives of the group (Figure 2D; **13** and **14**) [61]. 

### 3.5. Phenolic Compounds

Phenolic compounds represent a large group of secondary metabolites, which play multiple roles in photosynthetic organisms, including in defense strategies and in communication with interacting species [62,63]. Phenolic substances are biosynthesized mainly by the phenylpropanoid pathway, having phenylalanine or tyrosine as precursors. In microalgae, knowledge about their biosynthetic pathways is still scarce, however, a recent study has shown that the enzymatic apparatus for key phenolic biosynthetic intermediates is well conserved in all major algal divisions, although the ability to biosynthesize some subclasses of phenolics seems to be restricted to some groups [62]. For instance, phenolic acids such as vanillic, ferulic, and coumaric acids are widespread. Flavonoids are frequent in green algae (e.g., quercetin and apigenin) and cyanobacteria, in which unusual structures have been reported (e.g., ononin from *Synechocystis*) (Figure 2E) [62]. 

### 3.6. Terpenoids

Terpenoids are a prominent family of organic substances biosynthesized from C_5_ precursors, which are present in all living organisms [64]. Substances belonging to this group can also act as allelochemicals. Examples are comnostins, extracellular antibacterial diterpenoids produced by cyanobacteria *Nostoc* sp. [65], and the sesquiterpene eremophilone (Figure 2F), produced by cyanobacteria *Calothrix* sp. and toxic to invertebrates [66]. 

### 3.7. Volatile Organic Compounds

A specific group of allelochemicals is the volatile organic compounds (VOCs). VOCs are a broad group of compounds that encompass substances from different chemical classes, ranging from small molecules such as methane, to terpenoids and compounds derived from PUFAs including hydrocarbons, aldehydes, and ketones [25]. With a complex classification, the World Health Organization (WHO) defines VOCs as having boiling point ranges that have to fall between 50 °C and 100 °C up to 240–260 °C [67].

VOCs produced by microalgae have been widely demonstrated to have different ecological functions, including allelopathy. Environmental factors such as light, temperature, nutrition, and abiotic stresses affect VOCs emission [68]. Functions such as defense mechanisms, stress response, intra- and inter-species communication, modulating predator-prey interaction, and affecting biochemistry, metabolism, and physiology processes are described. These emissions from microalgae occur in vivo, in cells undergoing senescence or apoptosis, or when they are perishing under predator attack [69,70]. The role and the possible impact that these compounds have has been extensively studied and, in the future, they could be exploited for industrial purposes [67].

## 4. Allelochemicals from Microalgae for Biopesticides

Allelochemicals are considered safer and eco-friendly alternatives for efficient pest management as they have different and diversified modes of action compared with synthetic pesticides and are more easily degradable and less toxic to non-target organisms [71,72,73,74]. The potential use of allelochemicals from microalgae as biopesticides has attracted much attention lately [19,74,75,76,77]. In this section, we will present and discuss the potential use of allelochemicals from microalgae for the biocontrol of insects, weeds, and phytopathogens.

### 4.1. Allelochemicals against Phytopathogens

Allelochemicals from microalgae are known for their activity against a large spectrum of microorganisms, as evidenced by several reports [78,79,80,81]. Examples include hapalindole T, an antibacterial alkaloid from *Fischerella* sp. [82], nostofungicine, a lipopeptide with fungicide activity isolated from *Nostoc* sp. [83], and eicosapentaenoic acid (EPA), an antimicrobial fatty acid from *Phaeodactylum tricornutum* [84]. For this reason, extracts from microalgae have been evaluated for their potential against phytopathogens with encouraging outcomes. 

Senousy et al. [85] evaluated the methanolic extracts of ten microalgal strains against phytopathogenic fungi. Among them, three stood out: the cyanobacteria *Anabaena* sp. HSSASE11 and *Oscillatoria nigroviridis* HSSASE 15, which were active against the fungi *Botryodiplodia theobromae* and *Pythium ultimum*, respectively, and the Chlorophyta *Dunaliella* sp. HSSASE13 which was active against *Fusarium solani* [85]. Similarly, Schmid et al. [86] assayed aqueous extracts from five microalgae for their activity against fungal phytopathogens. The Chlorophyta *Scenedesmus obliquus* showed the best results, inhibiting *Sclerotium rolfsii* by 32% [86]. Another Chlorophyta, *Chlorella vulgaris*, was active against the phytopathogenic bacteria *Stenotrophomonas maltophilia*, which affects potato and green vegetables. The researchers evaluated three types of extracts: aqueous, ethanolic, and methanolic. Only the latter was active against the bacteria [87]. 

Interestingly, microalgae can also be active against phytopathogenic nematodes. Suspended algal cultures from *Scenedesmus obliquus*, *Chlorella vulgaris*, and *Anabaena oryzae* were able to inhibit the root-knot nematode (*Meloidogyne incognita*), a pathogen for banana cultures [88]. 

The activity of microalgae extracts against phytopathogenic organisms is frequently attributed to metabolites such as phenolics, alkaloids, and peptides [86,87,89]; however, few have conducted a thorough investigation to identify these compounds. In the study of Senousy et al. [85] mentioned above, the authors observed a positive correlation between the activity of the microalgal extracts against *Fusarium solani* and their phenolics and flavonoid content. These results indicate the probable chemical class of the bioactive substances. Phenolic substances also seem to be responsible for the antifungal activity of *Nannochloropsis* sp and *Spirulina* sp. Extracts composed of free phenolic acids from both microalgae exhibited significant inhibitory activity of *Fusarium* complex fungal pathogens. Both extracts had chlorogenic acid, a cinnamic acid derivative, (Figure 3; **21**) as their major component [90]. In another study, a phenolic substance isolated from culture filtrates of the cyanobacteria *Calothrix elenkinii* was reported as bioactive against the oomycete *Pythium debaryanum*. This substance, identified as 3-acetyl-2-hydroxy-6-methoxy-4-methyl benzoic acid (Figure 3; **22**), induced multiple morphological abnormalities in the hyphae of the microorganism [91]. 

Even though the antifungal mechanisms of the phenolic compounds mentioned above are not known, mechanistic studies on cinnamic and benzoic acid derivatives have been reported in the scientific literature. The antifungal activity of trans-cinnamaldehyde is related to the biosynthesis of cell wall components (glucan and chitin) [92,93]. In addition, methyl trans-cinnamate acts as an inhibitor of tyrosinase enzymes, which makes it interesting as a food anti-browning agent, but it also presented antimicrobial activity against bacteria and fungi [94]. Moreover, benzoic acid and its derivatives generally have multiple effects on fungal cells such as, for example, against *Alternaria solani* and *Eutypa lata* phytopathogens and other fungal genera. Several authors have discussed the fact that these phenolic substances generally affect cell respiration by altering cytochrome pathways, causing energy reduction by unbalancing intracellular benzoate levels, and inducing intracellular acidification, leading to protein aggregation and oxidative stress, for example. It is important to highlight that the effect of antifungal action depends on the concentration and molecular arrangement of these compounds [95,96,97].

### 4.2. Allelochemicals as Herbicides

The focus on the herbicide potential of metabolites from microalgae has also had some promising results. Allelochemicals identified as having phytotoxic activity belong to different chemical classes, but most of them were detected in cyanobacteria. Examples are depicted in Figure 4 and will be explored in this section. This is the case for cyanobacterin (Figure 4; **23**), a phenolic substance isolated from *Scytonema hofmanni* in 1982. The substance was demonstrated to be a potent inhibitor of the photosynthetic electron transport in photosystem II. The inhibition of photosynthesis is a potential mechanism of herbicides [98,99]. The peptide nostocyclamide (Figure 4; **24**), from *Nostoc* sp., and the alkaloids fischerellins (Figure 4; **26**), from *Fischerella*, were also shown to inhibit photosystem II [99]. 

The peptidic cyanobacterial toxins, the microcystins, on the other hand, are inhibitors of serine/threonine protein phosphatases, which results in cell growth suppression. They are phytotoxic due to their negative effects on plant physiology [99,100]. These substances, however, also represents a risk for human health and can bioaccumulate in vegetables, which hinders their application as herbicides [100]. Another substance with cell growth inhibitory activity is ambiguine D isonitrile (Figure 4; **25**), isolated from *Hapalosiphon* spp. This indole alkaloid was shown to suppress lettuce growth, an effect probably related to cell death induction by the overproduction of reactive oxygen species (ROS) and mitosis inhibition. Its potential as an herbicide remains to be further investigated [101]. 

Other substances with cell growth inhibitory activity may also exert herbicidal effects. For instance, cryptophycins (Figure 4; **27**), polyketides from *Nostoc* strains, which suppress microtubule polymerization. Methanolic extracts of a cryptophycin producer *Nostoc* strain hindered the growth of the stems and, in particular, the roots of a grass mixture [102], an effect that may be related to the cryptophycin content. Microalgae, especially cyanobacteria, are a rich source of cell division inhibitors, which have been primarily assessed for their antitumor or antimicrobial properties. These compounds, however, could also be useful as herbicides and represent an almost unexplored research field [99]. 

Metabolites from eukaryotic microalgae have been scarcely investigated for their herbicidal activity. However, some studies can be found, for example, VOCs from the green microalgae *Chlorella vulgaris* have evidenced a phytotoxic effect against barley. The mixture of volatiles consisted of hydrocarbons, carboxylic acids, aldehydes, alcohols, and ketones [103]. 

### 4.3. Allelochemicals as Insecticides

Allelochemicals produced by microalgae may be useful in the defense against plant predators. Thus, microalgae have been proposed as a source of allelochemicals with insecticide properties [104,105]. The good larvicidal performance of microalgae has also stimulated new technologies, such as the exploitation of the insecticide properties of *Chlamydomonas reinhardtii* extracts in the development of preparations based on nano/microparticles. The extract of the microalgae combined with microparticles of zinc oxide was able to double the mortality of mealworm beetle larvae (*Tenebrio molitor*) compared to zinc oxide alone [106]. 

**Figure 5:**
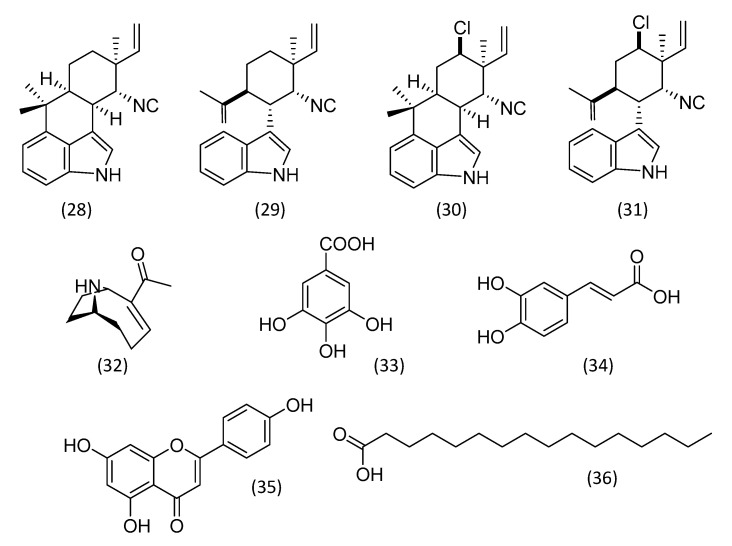
Microalgal substances with larvicidal or insecticidal activity. 12-*epi*-hapalindole J isonitrile (**28**), 12-*epi*-hapalindole C isonitrile (**29**), hapalindole L (**30**), 12-*epi*-hapalindole E isonitrile (**31**), anatoxin- A (**32**), gallic acid (**33**), caffeic acid (**34**), kaempferol (**35**), and *n*-hexadecenoic acid (**36**).

Biofilm-forming cyanobacteria showed defense mechanisms against insects that hinder feeding, such as the formation of colonies, filaments, mucilage, spines, and production of allelochemicals. The chemical compounds produced by their biofilm is considered more effective than other defense mechanisms. *Fischerella* strain 43,239 was selected in a cyanobacterial screening for insecticidal activity against the larvae of dipteran *Chironomus riparius* (harlequin fly). The toxic active fraction from *Fischerella* 43,239 had its chemical profile assessed by mass spectrometry, which provided evidence of the presence of indole compounds, considered possible bioactive substances [107]. Later, another study with *Fischerella* 43,239 identified indole alkaloids (Figure 5; **28**–**31**) with insecticide activity against the larvae of *Chironomus riparius*. Among the isolated hapalindoles, 12-*epi*-hapalindole J isonitrile was able to increase the larvae mortality at 26 μM within 48 h [108].

Extracts from cyanobacteria were tested against larvae of *Aedes aegypti*. Among the samples tested, *Microcystis aeruginosa* 205 and *Anabaena circinalis* 86 were the most active. The fractionation of these extracts showed the presence of anatoxin A and microcystins, which were toxic for *Aedes aegypti* larvae [109]. Microcystins are cyclic peptides isolated from cyanobacteria that exhibit high toxicity to insects [110]. These compounds were shown to form lesions in the midgut epithelial cells of *Aedes aegypt* larvae [111]. Anatoxin- A (Figure 5; **32**) is another insecticidal secondary metabolite from cyanobacteria that acts as an acetylcholine analogue. The action mechanisms of these compounds is similar to organophosphate and carbamate insecticides; the inhibition of the acetylcholinesterase enzyme (AchE) thus disrupts the nervous system of the insects [112]. An extract of cyanobacteria rich in anatoxin- A exhibited potential insecticide activity against *Nauphoeta cinerea* cockroaches. It is speculated that the compound caused cholinergic intoxication affecting the insect locomotion [113]. 

The insecticide potential of *Amphora coffeaeformis* (diatom) and *Scenedesmus obliquus* (Chlorophyta) were investigated against *Culex pipiens* mosquito larvae. The acetonic extract of these microalgae showed larvicidal activity. Among them, the extracts of *Amphora coffeaeformis* was the most active (LC_50_ = 513.63 μg/mL), while the lethal concentration of *Scenedesmus obliquus* was 855.66 μg/mL. The analysis of the extracts by HPLC-DAD detected the presence of phenolic compounds such as benzoic acids (gallic acid, methyl gallate, and syringic acid), cinnamic acids (caffeic acid and cinnamic acids), and flavonoids (taxifolin, kaempferol, and naringenin) [114]. The insecticidal properties of some of the phenolic compounds identified have already been described. Gallic acid (Figure 5; **33**) affects the growth of *Spodoptera litura* (tobacco cutworm) larvae and is toxic to *Aphis craccivora* (an aphid) [115,116]. Caffeic acid (Figure 5; **34**), a cinnamic acid derivative, inhibits the digestive protease of *Helicoverpa armigera* (polyphagous pest) [117]. Moreover, kaempferol (Figure 5; **35**) was able to cause 82% mortality of *Mythimna separata* (pest of cereal crops) in the concentration of 1 mg/mL [118]. 

Unsaturated fatty acids were also detected in *Amphora coffeaeformis* and *Scenedesmus obliquus* extracts by gas chromatography coupled with mass spectrometry (CG/MS). Fatty acids can also act against insects. Hexadecanoic acid methyl ester, 7- hexadecanoic acid methyl ester, and 9-octadecenoic acid (Z)-methyl ester were detected as major compounds in *Amphora coffeaeformism*, while in *Scenedesmus obliquus* extract (Z)-9-octadecenoic acid methyl ester, hexadecanoic acid (Figure 5; **36**) methyl ester, and 7-hexadecenoic acid methyl ester were the major compounds. It is speculated that the high content of phenolics compounds and unsaturated fatty acids in these extracts are responsible for their larvicidal activity. [114].

A study described unsaturated and saturated fatty acids as being responsible for the insecticidal activity of ethanolic extracts from microalgae against *Spodoptera littoralis* (cotton leafworm) larvae [119]. The bioassay-guided fractionation of chloroform and methanol extracts from *Chlorella* sp. showed that fatty acids (*n*-hexadecanoic acid, di-*N*-octyl phthalate, 1-hexacosanol, oleic acid, and 1-docosene) are among the larvicidal compounds in the active fractions. Some fatty acids detected in these fractions had their insecticidal activity reported [120]. Additionally, another study described *n*-hexadecanoic (Figure 5; **36**) acid as a potent larvicidal against *Culex quinquefasciatus*, *Anopheles stephensi*, and *Aedes aegypti* mosquitoes [121]. Fatty acids have also been evaluated in vitro against *Melanaphis sacchari* (an aphid) Zehntner larvae. Among them, linoleic acid showed 85% of mortality rate (LC_50_ = 1181 ppm) [122]. 

The possible mechanism of larvicidal activity of fatty acids was investigated by Perumalsamy et al. [123]. The authors evaluated some fatty acids for two common modes of insecticidal activity: the inhibition of AchE and the interference with the octopamine signaling pathways (cAMP level enhancement). While linoleic and linolenic acids were able to both inhibit AchE and enhance cAMP levels, palmitic and oleic acid inhibited AchE and had no significant effects on cAMP levels. Elaidic and arachidic acids where ineffective as AchE inhibitors but considerably enhanced cAMP levels. These results indicate that linoleic and linolenic acids might have a dual mode of action [123]. 

## 5. Safety and Toxicity of Allelochemicals and Synthetic Pesticides 

The herbicide resistance (HR) plant list continues to grow and is listed on the site: www.weedscience.org (accessed on 1 May 2023) [124]. New herbicides and pesticides are necessary to feed the world’s population and even more so as pests become resistant to currently available pesticides. Pesticides are, by nature, toxic and the challenge is to increase their specificity and reduce their persistence. Pesticides can cause adverse effects on the quality of the agricultural products they are used to protect. Pesticide residues can produce long-term adverse effects on the health of humans and animals and the stability of ecosystems by poisoning water sources. These chemicals can be the cause of neurotoxicity, endocrine disruption, Parkinson’s disease, cancers, and type 2 diabetes [125]. For instance, organochlorine pesticides, used in agriculture since the 1950s, are listed as a persistent organic pollutant (POP) and forbidden in most countries. Nevertheless, high concentrations of these pesticides and their transformation products (TPs) and degradation products, such as dichlorodiphenyltrichloroethane (DDT), are very persistent and have long half-lives. All of them are still detected in agricultural soils [126]. Modern formulations, called current-use pesticides (CUPs), are relatively safe to non-target species. The objective of modern formulations is to avoid persistent compounds that bioaccumulate and reduce their non-target toxic properties. However, they are capable of migrating over long distances and CUPs have been reported in air and surface water samples in the Arctic [127]. 

Allelochemicals as a source of biopesticides have been studied in greater detail from plants than from microalgae. However, several compounds found in plants with potential use as biopesticides are extensively present and described in microalgae, such as organic acids, esters, alkaloids, flavonoids, and terpenoids [19,76,128]. The multi-kingdom effects of some secondary allelopathic plant metabolites are known. Despite the original definition, that addresses plant–plant interactions, several compounds exhibit allelopathy and toxicity to other organisms. Currently, the term allelopathy is now used in this broader context by the International Society of Allelopathy and has been since the 1990s [129]. 

Plant allelochemicals typically present partial water solubility enabling easy application without surfactants and some of them are known to have similar modes of action to synthetic herbicides. Non-halogenated molecules decrease the environmental half-life while preventing soil accumulation. They have eco-environmental chemical structures with a higher nitrogen and oxygen content [130]. Modern technologies allow for their effective use and management and confer fewer environmental problems in soil due to the faster degradability of allelochemicals. However, rapid degradation of one of the chemical groups can decrease the compound’s bioactivity [131]. 

The diversity of allelochemicals makes them promising targets for discovering novel, specific target sites in plants or other organisms. Besides this, allelochemicals are also characterized by multi-site action, which differs from the synthetic pesticides, and affords them high specificity. In the case of synthetic herbicides, before they are licensed for use, they are tested to evaluate that their phytotoxic activity is in the range between 10^−5^ and 10^−7^ M. The chemical characterization of the molecular structure, the study of the mode/s of action, and their half-life in soil need to be established. It is also necessary to evaluate their influence on microbial ecology and other organisms, their potential toxic properties on human health, and the economic viability of their production on commercial scales [132]. Allelochemical toxicity depends on the concentration and compounds involved. Several secondary metabolites of other natural substance groups from plants and microalgae, such as glycosides, polyphenols, polysaccharides, terpenes, flavones, and alkaloids, have shown to be ecologically safe and biodegradable [133,134,135,136]. Triketones are one of the most important herbicide classes developed from an allelochemical triketone. These compounds inhibit invasive plants. Another example is cryptophycin-1, a depsipeptide from the cyanobacterium *Nostoc* sp. Cryptophycin-1 has an antiproliferative and antimitotic activity, inhibiting the cell cycle during the metaphase of mitosis, mainly in yeasts of the genus *Cryptococcus* [74,137].

## 6. Microalgae Allelochemicals: Gaps and Opportunities

Even though the prospects for the application of microalgal allelochemicals as biopesticides seem promising, there are still many knowledge gaps about these compounds. For instance, the action mechanisms of most of them is poorly understood. Some allelochemicals are known to have modes of action similar to synthetic pesticides, such as the insecticidal compound anatoxin A (inhibition of acetylcholinesterase) and the herbicidal molecule cyanobacterin (inhibition of photosystem II). However, many others are likely to exhibit new mechanisms and multi-target actions, which should be elucidated [132]. 

Moreover, their persistence and biodegradation in the environment needs further study. Once released into the soil, allelochemicals are more readily microbially degraded than xenobiotic persistent synthetics compounds. Their biodegradation depends on the soil composition and on the structure and population of the soil microbial community. Most assays conducted to investigate pesticide properties of allelochemicals are conducted under artificial laboratory conditions. Thus, to further estimate their potential in agricultural applications and to assess their environmental impact, in-field bioassays are necessary [129,131]. Studies on their possible toxic effects to non-target organisms, including humans, are also mandatory. These steps are important for any biopesticide candidate and require a great amount of interdisciplinary research [129]. 

The research and application of plant allelochemicals in agriculture is undoubtedly far more advanced than those from microalgae. However, concerning the production process, microalgal allelochemicals may have multiple advantages. Microalgae outdoor cultivation systems can be implemented in non-arable lands employing wastewater from agricultural or industrial activities, thus avoiding competition with food production. Their cultivation is also possible in closed bioreactors. In this case, the most important advantage is the possibility of having good control of the chemical and physical conditions of the whole process, which enables a high reproducibility and high yield [30,138]. The mass cultivation of microalgae for the production of food and chemicals on a commercial scale has already been established. The carotenoid, astaxanthin, for instance, which can be obtained from different microalgae, annually moves approximately 2 billion dollars in the global market [138,139,140]. For the industrial production of astaxanthin, *Haematococcus pluvialis* is cultivated in a two-step process. In the first stage, the microalgae grow in photobioreactors with a culture-rich medium for biomass accumulation. The second stage, in which the production of the carotenoid is induced by nutrient starvation, can be carried out in larger bioreactors or in open ponds. This process is energy-intensive and costly; however, improvements have been intensively researched and their implementation can substantially reduce the prices of the product [140,141].

Thus, since it is already possible to achieve bioactive substances from microalgae on a large scale, the obtaining of other compounds, such as allelochemicals, employing similar biotechnological processes can be envisaged. Nevertheless, the costs of biopesticide production through microalgae cultivation are still higher than the production of synthetic pesticides. However, the production of microalgae-derived biopesticides could be integrated with the production of biofuels and other valuable compounds within a biorefinery concept. This integrated approach is expected to lower the overall costs and enable better energy utilization as well as a reduction in the generation of residues [30,142,143,144,145]. Synthetic biology also has the potential to modify microalgal allelochemicals to improve activity, increase productivity and decrease non-specific toxicity [146].

The literature shows progress is being made in developing natural products from microalgae as biopesticides. Studies are in progress to reach commercial biopesticides from microalgae; in the future, they can complement or be an alternative to synthetic pesticides. At the moment, several promising less toxic and biodegradable allelochemicals have been identified and are worthy of further investigation into their application.

## 7. Perspectives

Much has been reported on the ecological effects of allelochemicals from plants and microbes but less so from algae and microalgae. All studies have highlighted the importance of the secondary “special” metabolism that is crucial for plant productivity and the biocontrol of pests in natural, agricultural, and anthropogenic environments. Studies on microalgae, while they have not received as much attention as agricultural crop plants, are equally important and present a new and promising low-cost source of bioactive compounds of increasing economic importance. As previously discussed, microalgae can be employed as bio-factories not only as a source of green energy but also a low-cost source of bioactive molecules, which can be useful for agriculture with minimal negative environmental impact. In this light, the future for using algae within a circular economic context is strengthened and the value of the algal production is increased by separating the differing components of their metabolism. The mechanisms of algal allelopathy require further study and specifically at the genetic level. It seems highly likely that gene editing with CRISPR will only increase the production of molecules of interest in a directed way. Given that secondary metabolism often occurs in response to a stress, be that competitive or environmental, genetic studies involving the algal holobiont (algae + bacteria + virus) and the role of interference RNAs will help us manage the algal bio-factory. The increased use of bioactive compounds from algae is promising. Here, we have described the potential of the interesting allelopathic compounds from microalgae. We have made the case for more intensive research on microalgae and hinted that microalgal bio-factories warrant industrial investment.

## Data Availability

No new data were generated in this review article.
